# Adverse effects of methamphetamine on vital organs of male rats: Histopathological and immunohistochemical investigations

**DOI:** 10.22038/IJBMS.2023.68573.15055

**Published:** 2023

**Authors:** Shahrzad Azizi, Reza Kheirandish, Shahriar Dabiri, Mina Lakzaee

**Affiliations:** 1 Department of Pathobiology, School of Veterinary Medicine, Shahid Bahonar University of Kerman, Kerman, Iran; 2 Pathology and Stem Cell Research Center, Pathology Department, Afzalipour Kerman Medical School, Kerman, Iran

**Keywords:** Brain, Histopathology, Immunohistochemistry, Male rats, Methamphetamine

## Abstract

**Objective(s):**

Methamphetamine (named crystal, ice, and crank), is a strong psychostimulant drug with addictive and neurotoxic properties. It is absorbed by various organs and induces tissue damage in abusers. Most METH studies have focused on the central nervous system and its effects on other organs have been neglected. Experimental investigations of animal models are used to provide significant additional information. We have studied the histopathological effects of methamphetamine in the brains, hearts, livers, testes, and kidneys of rats.

**Materials and Methods:**

Methamphetamine (0.5 mg/kg) was administered subcutaneously for 21 days. Immunohistochemistry was carried out with markers including glial fibrillary acidic protein (GFAP) for reactive astrocytes, vimentin as an intermediate filament in different cells, and CD45 marker for the detection of reactive microglia in the brain. Also, some samples were taken from livers, kidneys, hearts, and testes.

**Results:**

Degenerative changes and necrosis were the most common histopathological effects in the liver, kidneys, heart, testes, and brains of rats treated with methamphetamine. Immunohistochemical analyses by vimentin and GFAP markers revealed reactive microglia and astrocytes with the appearance of swollen cell bodies and also short, thickened, and irregular processes. Moreover, the number of CD45-positive cells was higher in this group. Reactive cells were more noticeable in the peduncles and subcortical white matter of the cerebellum.

**Conclusion:**

Our results showed the toxic effects of methamphetamine on the vital organs and induction of neurotoxicity, cardiomyopathy, renal damage, and infertility in male rats. We could not attribute observed hepatic changes to METH and further evaluation is needed.

## Introduction

Methamphetamine (METH), known as ecstasy, is a strong psychostimulant drug with addictive and neurotoxic properties (1). METH was synthesized by the Japanese pharmacologist Dr. Nagayoshi Nagiai (1888) by reducing ephedrine with hydriodic acid and red phosphorus. METH is also referred to by some conversational names such as crystal, ice, speed, whiz, and crank (2). METH abuse is considered to be a social and global problem because of its detrimental consequences including crime, the cost of treatment, and adverse psychiatric and socioeconomic outcomes. It is the second most frequently used drug after cannabis (3). This drug is associated with euphoria, a sense of energy, reduction of anxiety, motivation, and psychomotor activity (4). Because of these effects, a tendency for METH abuse is developing among young adults in most parts of the world (5).

METH is available in different forms including powder, paste, and crystals. Routes of utilization are sniffing, oral ingestion, pulmonary inhalation, and injection (6). Smoking is the most common method of administration (7). METH abuse increases the risks of HIV and hepatitis viral infections, high blood pressure and temperature, pulmonary hypertension, periodontal diseases, adrenergic activities, cerebrovascular disturbances, circulatory collapse, and renal failure (8).

This drug is distributed rapidly in the various organs and induces tissue damage in the abusers (9). Most studies of METH have been focused on the CNS, and its effects on other organs are not obvious (8). There are limitations in human postmortem evaluations such as the limited number of human cases, tissue sample accessibility, and the complexity of sample preparation. Therefore, experimental studies using animal models can provide important information. Also, unlike animal sampling, whose condition at death can be controlled and influenced, human specimens can only be collected after natural death has occurred, and the time of death is not controllable. In this research, histopathological effects of METH have been studied in the brains, hearts, livers, testes, and kidneys of rats. Immunohistochemical techniques were also performed using specific brain markers. 

## Materials and Methods

***Experimental animals***

This study was approved by the Ethics Committee of Shahid Bahonar University of Kerman. In the present study, 10 adult male rats (200–250 g) were used. The animals were housed in clear plexiglass cages under a 12/hr light-12/hr dark cycle with free access to standard food and fresh water. The animals were randomly divided into METH and control groups (n=5 rats in each group). 

***Drug preparation***

METH (purity˃98%, donated by Iran’s Drug Control Headquarters) was freshly dissolved in sterile physiological saline solution (0.9% NaCl) before usage and administered subcutaneously (over the shoulders, into the loose skin over the neck) at a dosage of 0.5 mg/kg for 21 days (10). Animals in the control group received saline solution only with the same dosage.

***Histopathological investigations***

After the last injection of METH, animals of both METH and control groups were euthanized by a high dose of anesthesia by using ketamine (Alfasan, Woerden, Holland) and xylazine (Alfasan, Woerden, Holland). Samples were taken from livers, kidneys, hearts, testes, and brains and fixed in 10% neutral buffered formalin for 48 hr. After fixation, the samples were processed according to routine histopathological procedures. Tissue paraffin sections of 5 μm thickness were cut and then stained with the hematoxylin-eosin method. The sections were studied using a light microscope (Nikon, Digital Sight DS-Fi2, Japan). In addition, Masson’s trichrome staining was used for the heart tissue. For the brain samples, immunohistochemical techniques were applied with antibodies against vimentin (IR630, Dako), GFAP (IR524, Dako), and CD45 (IR751, Dako). 

Histomorphometric analysis was conducted on testicular tissue. In each sample, four parameters including Johnsen’s score (JS), spermatogenesis index (SPI), meiotic index (the ratio of round spermatids to primary spermatocytes), and seminiferous tubule diameter were evaluated. The meiotic index was investigated in 100 seminiferous tubules. For seminal tubule diameter, the 10 smallest and roundest tubules were chosen and measured by a graded microscopic lens. In addition, for JS, the seminiferous tubules were graded according to the semi-quantitative JS. In this system of classification, 100 seminiferous tubules in each section of the testicular tissue were considered and a score from 1 to 10 was given to each tubule based on Johnsen (11). The mean of 100 scores was considered JS (12). To evaluate the SPI index, seminiferous tubules with spermatozoa inside their lumen were considered as tubules with a positive spermatogenic index, and empty ones without spermatozoa in the lumen were considered as having a negative spermatogenic index. For this parameter, a total of 100 seminiferous tubules were counted in different groups (13).

***Statistical analysis***

The results were analyzed by SPSS 17.0 (SPSS Inc., Chicago, IL, USA). Independent Student’s t-test was used for investigated parameters to compare the control and methamphetamine groups. Data are expressed as mean standard deviation (mean±SE). *A P*-value less than 0.05 was considered as a statistically significant different level.

## Results

Methamphetamine administration for 21 days induced histopathological damage such as degenerative changes and necrosis in the evaluated organs of all METH-treated rats. The results are explained separately in different sections as follows. 

***Histopathological and immunohistochemical findings of METH-treated brains***

In the control group, most of the neurons in the cerebral cortexes had a healthy appearance with distinct central nuclei, marked nucleoli, and light blue cytoplasm. Only a few nerve cells had condensed and dark nuclei with hyperchromatic cytoplasm. 

**Figure 1:**
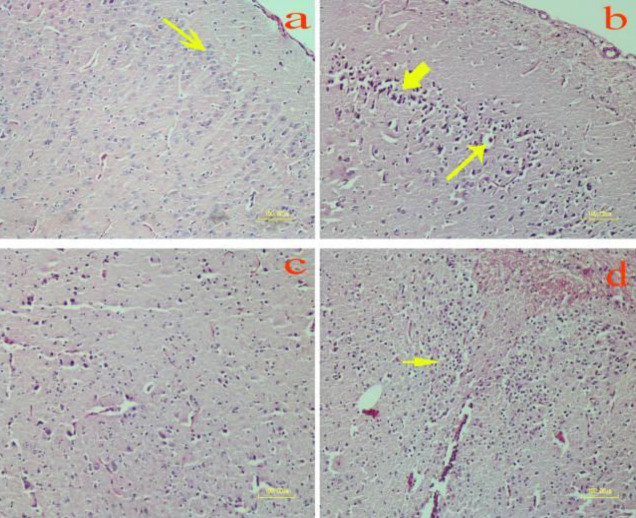
Effect of methamphetamine on the brain of rat. a) Control group: Healthy neurons with light basophilic color; Methamphetamine group: b) Necrotic neurons in the cerebral cortex are shrunken and hyperchromic (thick arrow). Spaces around the neurons are dilated due to edema (thin arrow); c and d) Gliosis (arrow) is diagnosed with marked hypercellularity compared with the control group (a)(H&E staining)

**Figure 2:**
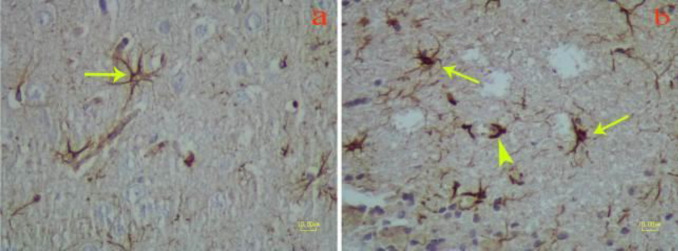
Immunohistochemistry on the brain of rat with GFAP marker. a) Control group: Long, smooth, and regular processes of normal astrocyte (arrow); b) Methamphetamine group. Astrocytes with hypertrophied cell bodies (thin arrows) and thick, irregular, wavy form and shorter processes in comparison with normal astrocytes in the control group (arrowhead)

In all the METH-treated rats, damaged neurons in the cerebral cortexes were identified with increasing cytoplasm eosinophilia and pyknotic nuclei. Brain edema was diagnosed with dilated perivascular and perineuronal spaces. A marked and obvious histopathological feature in this group was increased hypercellularity due to the occurrence of gliosis (Figure 1). The reactivity of astrocytes was demonstrated with GFAP, a common histological marker for astrocytes. Immunohistochemistry staining depicted increased GFAP expression that was more intense in the subcortical white matter of the cerebellum than in other parts of the brain such as the cerebral cortexes. Reactive astrocytes had hypertrophic soma. Their processes were thicker, irregular, wavy in form, and shorter than the healthy astrocytes. In the control group, healthy astrocytes were star-shaped with slender processes (Figure 2). Moreover, GFAP-immunoreaction was observed in the radial glial processes under the meninges in both control and METH groups.

**Figure 3:**
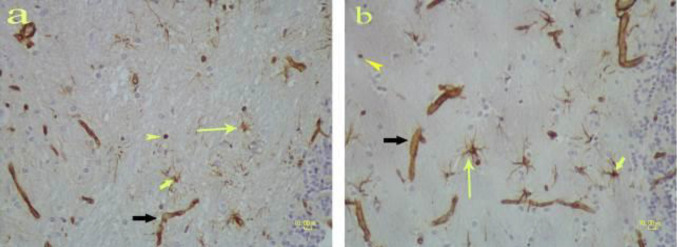
Vimentin immunostaining on the brain of rat. b) Methamphetamine-treated brain. Microglia (yellow thick arrow) and also stellate astrocytes have hypertrophic cell bodies and thicker processes (yellow thin arrow) compared with the control group (a). In both groups, vascular walls (black arrow) and some spherical cells (arrowhead) show positive reactions with vimentin

Vimentin-immunoreactivity was considered another sign of astrocyte activation. It was detectable in the submeningial areas and blood vessels in the control and METH groups but more intensive immunoreactivity was observed in the METH group in the mentioned areas. In both groups, three types of cells showed vimentin positivity, and one of the positive cell types was astrocytes. In the METH group, hypertrophy of astrocytes was identified with increased vimentin expression, especially in the subcortical areas of the cerebellum (Figure 3). The second type of vimentin-positive cells was rod-shaped microglia, which showed a hypertrophied appearance in the METH-treated rats compared with the control group. The third type of positive cells had a spherical morphology with higher numbers in the METH group. Also, some aggregations of strongly reactive astrocytes were found in this group. 

**Figure 4:**
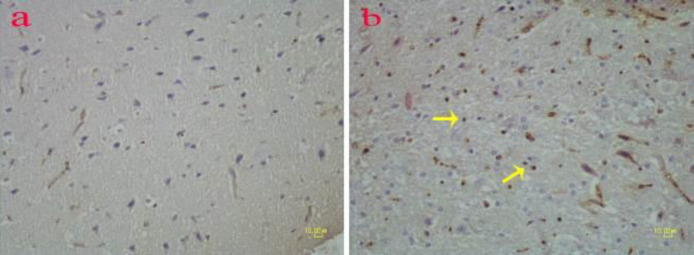
Immunohistochemical detection of CD45 marker on the brain of rat. The number of CD45 positive cells (arrows) in the methamphetamine group (b) is higher than in controls (a)

We used CD45 antibody in order to detect microglia reaction but this marker is not completely specific for microglial cells. The METH group revealed an increase in CD45-positive cells in comparison with the control group. The reactive cells were dispersed diffusely in different parts of the brain but the number of cells was greater in the subcortical areas of the cerebellum, cerebellar peduncles, and pons. CD45-positive cells were observed in two forms including spherical (Figure 4) and rod-shaped cells. 

***Histopathological findings of METH-treated hearts***

Histopathological characteristics of the hearts in the control group revealed regular arrangements of normal cardiac fibers. The sarcolemma of myocytes was normal and no fractures, degenerative changes, or hyalinization were observed. The nuclei of cardiac myocytes were vesicular and located in the central areas of the myocytes.

**Figure 5:**
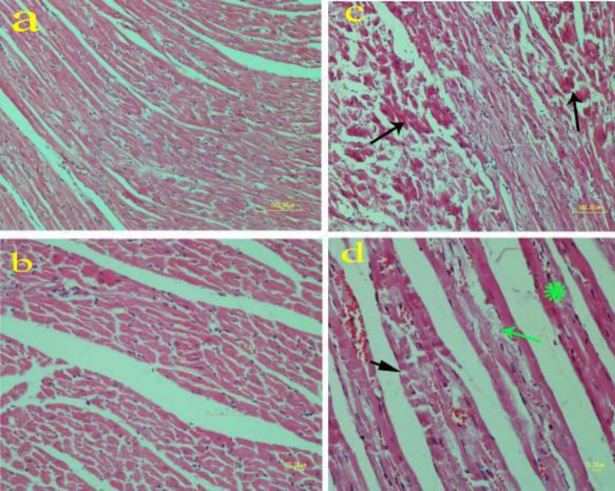
Effect of methamphetamine on the heart of rat. a, b) Control group: cardiac fibers are regular and no abnormal changes are present; c, d) Methamphetamine group. Disorganization and necrosis of cardiac fibers are characterized by hyalinization and pyknotic nuclei (arrows) in Figure c. Sarcolemma disruption (black arrow), blood congestion, interfiber edema (green arrow), and hyalinization (asterisk) can be seen in Figure d. (H&E staining)

**Figure 6:**
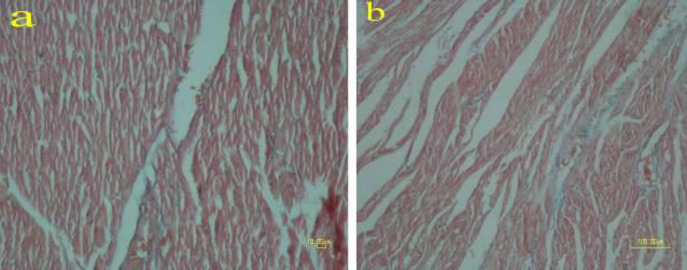
Masson trichrome staining on the heart of rat. No myocardial fibrosis has occurred in the methamphetamine group (b) compared with the control group (a)

In contrast, in the METH group, cardiac tissue showed general disorganization. Irregular arrangements and mild widening of the myocardial fibers were observed. The sarcolemma of the cardiomyocytes had become fragile and lost its integrity. The myocytes revealed degenerative changes and Zenker’s necrosis. Degeneration was diagnosed by vacuolization of the cytoplasm. A few bizarre-shaped nuclei were present in some cells. Zenker’s necrosis was identified with hyalinization of myocardial fibers. Interfibrillar space had been increased due to interstitial edema (Figure 5). No evidence of fibrosis was observed by H&E or Masson’s trichrome staining (Figure 6).

***Histopathological findings of METH-treated kidneys***

In the control group, histopathological findings of the kidneys showed normal structure in the glomeruli and distal and proximal tubules. Renal tubules were lined with a simple cuboidal epithelium. Epithelial cells had light eosinophilic cytoplasm, vesicular nuclei, and also visible brush borders in the proximal tubules. No signs of glomerular atrophy, tubular degeneration, necrosis, or infiltration of inflammatory cells were observed. 

**Figure 7:**
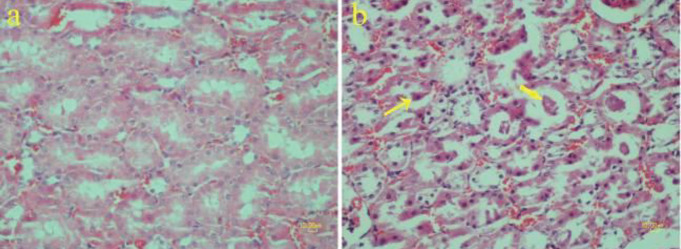
Effect of methamphetamine on the kidney of rat. a) Normal structure is obvious in the control group. The tubular epithelial cells have light pink cytoplasm and vesicular nuclei. b) The methamphetamine group shows necrotic changes including hypereosinophilic cytoplasm and nuclei pyknosis (thin arrow) in the tubular epithelium. Also, proteinaceous substances are present in the lumen of some tubules (thick arrow) (H&E staining)

**Figure 8:**
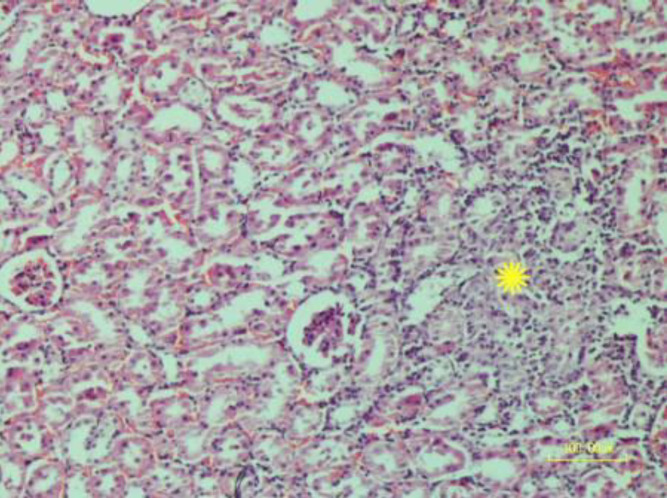
Mononuclear inflammatory cells are infiltrated in the renal interstitial tissue of rat. (asterisk). In addition, tubular epithelium exhibits necrotic changes (H&E staining)

In the METH-treated rats, some glomeruli were atrophied and the urinary space of Bowman’s capsule was dilated. A complete absence of some glomeruli was observed. The histopathological changes of the epithelium of the tubules varied from the presence of clear vacuoles in the cytoplasm to necrosis in both proximal and distal tubules. The necrotic epithelial cells had pyknotic nuclei and deeply red cytoplasm. The lumen of some tubules was filled with eosinophilic substances (Figure 7). Mononuclear cells, especially lymphocytes, had infiltrated into the interstitial tissues of the renal parenchyma (Figure 8).

***Histopathological and morphometric*** ***findings of METH-treated testes ***

Testicular tissue of the control group showed normal seminiferous tubules. Different stages of spermatogenic cells including spermatogonia, spermatocytes, and spermatids were normal. The loose connective tissue between the seminal tubules comprised fibroblasts, blood vessels, and Leydig cells.

**Figure 9:**
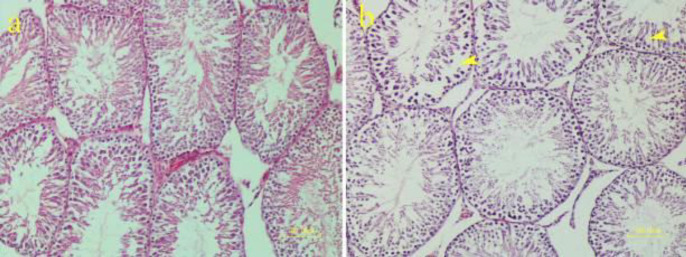
Effect of methamphetamine on the testicular tissue of rat. a) Normal morphology of seminiferous tubules in the control group, b) disorganization, depletion of germinal cells, and increasing spaces (arrowheads) between epithelial cells in the methamphetamine group (H&E staining)

In all rats treated with METH, spermatogenesis reduction was seen to different degrees. In some tubules, lack of spermatids and spermatozoa was observed. The germinal epithelium was disorganized. Spaces between spermatogenic cells had been increased due to disruption of the integrity of the germinal layer (Figure 9). Histomorphometric parameters, including the percentages of spermatogenesis, diameters of seminiferous tubules, meiotic index, and Johnsen’s score, showed a remarkable decrease compared with the control group (*P*<0.001)(Table 1). 

***Histopathological findings of METH-treated livers***

**Figure 10:**
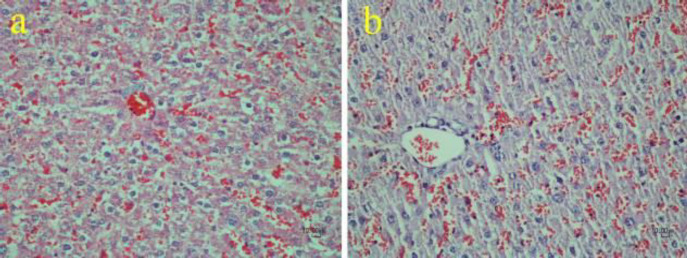
Effect of methamphetamine on the liver of rat. Congestion and vacuolar degeneration are observed in both the control (a) and methamphetamine (b) groups

In the livers, histopathological alterations included congestion and vacuolar degeneration in the cytoplasm of hepatocytes in both the METH-treated rats and the control group. No obvious microscopic changes were present in the two groups (Figure 10).

## Discussion

Methamphetamine (N-methyl-O-phenyl isopropylamine) belongs to the phenethylamine and amphetamine group of psychoactive drugs. Physical and mental disorders associated with METH are mainly attributed to its effects on the monoaminergic system within the central and peripheral nervous systems. This synthetic drug overturns the neurotransmitter transport pathway and increases the extracellular concentrations of dopamine (DA), norepinephrine (NE), and serotonin (5HT). Enhanced concentration of dopamine in the brain causes a euphoric feeling, excitation, and alertness in METH abusers (14). The 5HT in the limbic system and hypothalamus increases oxytocin which is responsible for the emotional effects of METH (15). In addition to neurological effects, METH exposure induces failure in the cardiovascular, urogenital, digestive, and hepatic systems (16). Despite its widespread popularity in recent decades, the adverse effects of METH on other organs far from the CNS have attracted little attention. 

In the current study, the toxic effects of methamphetamine (0.5 mg/kg/day) have been investigated histopathologically in the brain, heart, kidneys, liver, and testes. Following an alternate approach in order to gain a better understanding of glial cell reactions, the immunohistochemical technique was performed using specific antibodies including glial fibrillary acidic protein (GFAP: for reactive astrocytes), vimentin (as an intermediate filament in different cells) and CD45 markers (detection of reactive microglia). Different markers can be used for immunohistochemical studies of the brain and each marker gives valuable and useful information. Histopathological results in the brain showed neuronal necrosis, gliosis, and the occurrence of edema in the cerebrum of the METH-treated group. We used the GFAP marker for the investigation of astrocytic gliosis. Astrocytic gliosis was associated with an increase in GFAP especially in the subcortical white matter of the cerebellum compared with other parts of the CNS. Reactive astrocytes revealed hypertrophic soma and thick, irregular processes in comparison with astrocytes in the control group. The GFAP marker is generally used for the evaluation of astrocyte reactivity (17). Indeed, the use of antibodies against GFAP in histopathological examinations has illustrated the existence of reactive gliosis as a response to brain injuries.

Our histopathological and GFAP immunohistochemical findings are consistent with previous studies. Lafuente *et al*. (18) examined METH neurotoxicity in chronic forced swim and METH groups of rats and showed that it was accompanied by neuronal damage in different parts of the brain. These researchers observed darkening and shrinkage of neurons, status spongiosis, and edematous neuropil. These changes were exacerbated in the METH-treated group under the influence of chronic forced swim stress. Also, marked activation of astrocytes was shown by GFAP immunohistochemistry in the experimental groups. Researchers (19) used GFAP immunoreactivity for the determination of morphological changes in astrocytes of METH abusers. They observed clasmatodendrotic astrocytes that were characterized by swollen cell bodies and beaded form processes in the IV, V, and VI layers of the cerebral cortex. Sharma and Kiyatkin (20) described the effects of acute METH intoxication (9 mg/kg) on morphological abnormalities of brain cells at temperatures of 23 °C and 29 °C in rats. METH-exposed brains showed expansion of the cortex, perineuronal edema, spongiosis, and pyknotic neurons in contrast to the control brains. GFAP immunopositivity for glial cell alteration was significantly more intense in the METH group than in the control rats. In another study conducted by O’Callaghan and Miller (21), GFAP expression was seen in rats exposed to METH after 21 days. 

GFAP up-regulation is a strong indicator for reactive astrocytes. It is an important marker of neurodegenerative disorders such as Alzheimer’s disease, Parkinson’s disease, and HIV-associated dementia (22). 

In the present study, METH-induced neurodegenerative lesions and neuronal necrosis in the cerebral cortexes of rats can be related to vasoconstriction. METH causes norepinephrine liberation from the axon terminals of noradrenergic nerves. Norepinephrine regulates α-1 noradrenergic receptors on pial arteries which induces vasoconstriction and ischemic damage of nerve cells in the cortex (23). It is shown that METH distributes throughout BBB and results in brain edema (24).

In the present study, vimentin was expressed in vascular structures, the subpial area, astrocytes, and some spherical to rod-shaped cells in both METH and control groups. Higher vimentin expression was observed in the METH-treated brains. Hypertrophic astrocytes and rod-shaped microglia were remarkable in the subcortical white matter of the cerebellum and cerebellar peduncles and scarce in the cortex or other regions of the CNS. 

In the literature, vimentin has been expressed in a variety of cells such as astrocytes and microglia. Vimentin is an intermediate filament mainly present in the mesenchymal cells (25). It is widely expressed in the embryonic stage and then replaced by GFAP during maturation (26). In the healthy adult brain, vimentin immunoreactivity is limited to the endothelial cells and subpopulations of glial cells (27). In some pathological conditions, vimentin up-regulation is considered an early sign of astrocyte activation (28). Graeber and Kreutzberg (29) stimulated microglia via facial nerve axotomy in rats and investigated immunocytochemical findings by electron microscopy. They observed vimentin at the intermediate filament sites and suggested that vimentin expression may assist as a marker for the recognition of activated microglia.

In the present study, the CD45 marker was used for the detection of reactive microglia. We observed some CD45-positive cells with spherical shapes and also a few cells with rod-shaped appearances. The number of CD45-positive cells was higher in the METH-treated rats compared with the control group. The reactive cells were dispersed in different parts of the brains but the white matter of the cerebellum, cerebellar peduncles, and pons revealed higher reactivity. We could not confirm exactly whether these positive cells were microglia. 

In the literature, different results are documented for CD45-positive cells. A variety of markers including histocompatibility complex I and II, CD68, CD45, and GLUT5 have been used for identification of cell hypertrophy, and proliferation and migration of microglia (30). CD45 is known as a leukocyte common antigen that is expressed on all nucleated hematopoietic cells. This marker plays a significant role in the regulation of immune responses (31). Researchers (32) used flow cytometry for the assessment of inflammatory cells following focal cerebral ischemia in mice. They found that CD45-positive cells could be subdivided into CD45-low and CD45-high cells for resting microglia, and activated microglia as well as peripheral leukocytes, respectively.

The neurotoxicity mechanism of METH is complex and involves multiple pathways. METH increases levels of extracellular monoamine neurotransmitters (dopamine, serotonin, and norepinephrine) by stimulating the sympathetic nerves (33). Dopamine is oxidized to quinone or semi-quinone, which produce reactive oxygen species (ROS) such as H2O2, OH-, and O2. Oxidative stress has a critical role in cellular toxicity (34). Mitochondria are the principal organelles in the production of ROS within nerve cells (35). METH disrupts the metabolism of mitochondria. Derangement in mitochondrial metabolism results in dopaminergic neuron toxicity due to inhibition of the Krebs cycle and electron transport chain (36), apoptosis induction in neurons, and activated astrocytes and microglia (8, 37).

***Kidneys***

We studied the detrimental effects of METH on kidneys. The METH-exposed kidneys revealed histopathological changes including glomerular atrophy, vacuolar degeneration, tubular necrosis, hyaline cast in the lumen of tubules, and interstitial nephritis. Previous research confirmed our pathological results. Researchers (16) studied the effect of pentoxifylline on METH-induced apoptosis in the kidneys of rats. Histopathological changes in the METH group were tubulointerstitial injury, a marked decrease of tubular epithelial cells, and increased TUNEL-positive cells. The activity of caspase-3 had been increased under the influence of methamphetamine. These researchers concluded that kidneys are an absolute target organ for METH-induced toxicity effects.

Ishigami *et al*. (38) conducted a myoglobin immunohistochemical study on 22 kidney samples obtained from human autopsy in which METH had been detected. Seventeen of the samples showed immunopositive myoglobin markers. Myoglobin-positive cases had higher blood METH than negative ones. Microscopic features in the affected kidneys included detachment of the tubular epithelium from the basement membrane, necrotic changes, and the presence of a hyaline cast in the tubular lumen. These researchers suggested METH poisoning could be diagnosed by the measurement of METH concentration in blood. Also, immunohistochemistry can use some markers, such as myoglobin, HSP70, 8-OH-dG, 4-HNE, and SOD, to detect poisoning with METH.

Researchers (39) applied immunohistochemical techniques in order to find a way to diagnose the acute or sub-acute effects of METH intoxication in kidneys. Also, renal function was evaluated by measuring the level of minerals, myoglobin, and creatinine phosphokinase (CPK) in the blood. METH was administered in both methods as a single dose (50 mg/kg/, IP) and repeated doses (10 mg/kg/day, IP) for 5 days. In single dose injection, ubiquitin immunoreactivity was enhanced in the renal tubules. Significant increases in creatinine and CPK, and decreases in K, Ca, and P were reported. Therefore, METH caused renal tubule injury that resulted in renal dysfunction. This renal injury might be related to leakage of CPK from muscles. In the case of repeated doses of METH, the immunoreactivity of 8-hydroxy-20-deoxyguanosine (8-OHdG) increased significantly. Thus, repeated METH administration may induce oxidative DNA damage. They believed that these results provided basic information for pathological investigations of the kidneys of autopsied cases.

Different mechanisms are mentioned for METH renal injury. Kidneys may be damaged following traumatic rhabdomyolysis, thrombotic microangiopathy, urinary tract obstruction, hypertension, and proximal tubule dysfunction (40). One of the remarkable mechanisms in cocaine and METH abusers is the enhancement of the sympathomimetic activity, which results in general vasoconstriction and ischemic destruction (41).

***Heart***

Cardiac failure is responsible for some METH-related deaths. We investigated cardiac lesions after 21 days of METH injection in rats. The subsequent microscopic findings included mild hypertrophy, irregular arrangement of myocardial fibers, sarcolemma disruption, cytoplasmic vacuolization, hyalinization, and interstitial edema. No evidence of cardiac fibrosis was demonstrated by H&E and Masson’s trichrome staining. Similar results such as hypertrophy, cellular breakup, contraction band necrosis, fiber disarray, and vacuolization, as well as fibrosis, are reported by other researchers. 

The most recent studies suggest that METH cardiac toxicity is induced by oxidative stress (42, 43). Researchers (44) investigated 100 cases of death related to methamphetamine toxicity. In this research, liver, gastric content, bile, urine, blood, and vitreous humor were examined by thin-layer chromatography, gas chromatography/mass spectrometry, and HPLC for toxicological analysis. Methamphetamine and amphetamine were detected in the urine and gastric contents. The most common histopathological features were reported as hypertrophy of myocardial fibers, atherosclerosis, and focal degeneration to necrosis. Methamphetamine toxicity was known to be the direct agent of death in all cases. They concluded that methamphetamine cardiotoxicity is one of the major causative factors in death.

Methamphetamine increases the release of catecholamines. Excess catecholamines stimulate alpha- and beta-adrenergic receptors. This stimulation leads to coronary vasospasm, ischemia, production of oxygen species, calcium overload, and mitochondrial alterations that are primary mechanisms in myocardial injury (45, 46). Commonly reported symptoms following methamphetamine use are chest pain, tachycardia in the short-term, hypertension, arrhythmias, coronary artery rupture, aortic dissection, and sudden cardiac death (47, 48). 

***Testes***

In recent years, the negative impacts of METH on the reproductive system have been studied because METH may be teratogenic and embryotoxic (49). In our study, spermatogenesis was reduced following METH administration. The germinal epithelium of seminiferous tubules was disrupted and disorganized. Spermatogenesis parameters including sperm production, seminiferous tubule diameter, meiotic index, and Johnsen’s score were significantly lower than the control group. Previous research supports our testes results. In a recent study (50), METH (5 ml/kg, IP) was associated with germ cell loss and decreases in spermatogenesis indices (tubular differentiation index, spermiogenesis index, repopulation index, and seminiferous tubule diameter) compared with the control group during 7 and 14 days of METH administration.

Researchers (51) described the toxic effects of the administration of METH (10 mg/kg) during courses of 15, 30, 60, and 90 days. Their results showed high sperm abnormalities, increasing oxidative stress markers and apoptotic index (*Bad/Bcl2 *expression ratio), and decreasing testosterone levels in the serum. They concluded that damage to the testes interferes with the normal functioning of the reproductive system and has adverse effects on male fertility. In another study (52), METH had reduced Leydig cells and the expression of androgen receptors in both round and elongated spermatids leading to impaired spermatogenesis.

It has been reported that METH increases testicular gamma-aminobutyric acid (GABA) concentration in rats. GABA contributes to Leydig cell proliferation and testosterone production. The hypothesis has been proposed that increasing GABA concentration is a compensatory response to the harmful effects of methamphetamine on Leydig cells (53). Another mechanism related to METH abuse has been described by Yang *et al*. (54) in two models, one designed to give short-term exposure and the other chronic exposure. They injected METH in different doses and courses into rats. The results showed low testicular index and sperm counts in the chronic model. The mRNA and protein expression of glucose transporter 1, hexokinase 1, and lactate dehydrogenase C were reduced in the chronic METH model. Their results also indicated that the glucose metabolite G-6-P had increased after short-term METH exposure but decreased in chronic exposure to METH. They suggested that glycolysis is reduced in the testes following the chronic injection of METH and leads to defects in spermatogenesis.

***Liver***

In our study, microscopic vacuolar degeneration and congestion were manifested in both METH and control groups without a noticeable difference. Therefore, we could not interpret these changes, and further examination is needed. However, previous studies have reported similar histopathological lesions in the livers of METH abusers (55, 56). 

A study (57) reported liver dysfunction, psychosis, hyperthermia, and rhabdomyolysis in a 41-year-old man, 6 hr after intravenous injection of METH. Histopathological examination of the liver biopsy showed hepatic necrosis and ballooning degeneration in the centrilobular zones.

Merchant *et al*. (58) described hepatic and pancreatic ischemia in a 35-year-old man, who had died as a result of a METH overdose. In autopsy, his liver had gross changes related to steatosis. The presence of macrovesicles in the cytoplasm of hepatocytes, enumerable necrotic bile duct hamartomas, and thrombi in the hepatic vessels were demonstrated histopathologically. Cerebellar congestion and acute pancreatitis were other reported lesions. They stated that widespread use of METH induces injuries in different organs as a result of vasoconstriction and subsequent ischemia.

The exact mechanisms responsible for METH hepatotoxicity are unknown. It is documented that METH increases ammonia levels in the plasma and brain which is correlated with hepatotoxicity (56). Recently, *in vitro* studies have shown that high concentrations of METH and other amphetamines lead to hyperthermia and hepatocyte injuries (59, 60).

**Table 1:** Effects of Methamphetamine (METH) on the spermatogenic parameters in rats (Mean±SE)

**Spermatogenic parameters**	**Control**	**Methamphetamine**	***P*** ***-*** **value**	
**Tubular diameter**	298.33 ± 1.52	281.67 ± 2.41	< 0.05	
**Spermatogenesis (%)**	97	83.33	< 0.05	
**Meiotic** **index **	2.95 ± 0.23	2.13 ± 0.24	< 0.05	
**Johnsen score**	8.76 ± 0.17	7.83 ± 0.14	< 0.05	
**Spermatogenic parameters**	**Control**	**Methamphetamine**	***P*** ***-*** **value**
**Tubular diameter**	298.33 ± 1.52	281.67 ± 2.41	< 0.05
**Spermatogenesis (%)**	97	83.33	< 0.05
**Meiotic** **index **	2.95 ± 0.23	2.13 ± 0.24	< 0.05
**Johnsen score**	8.76 ± 0.17	7.83 ± 0.14	< 0.05

## Conclusion

A review of the literature shows that the harmful effects of METH on the CNS have been extensively studied, whereas injuries to other organs have received less attention. The current study investigated the consequences of METH injections for the brain as well as for other vital organs such as the kidneys, heart, liver, and testes in rats. In this study, we could not attribute observed hepatic changes to METH. Our results demonstrate that the repeated administration of METH is harmful to vital organs and induces neurotoxicity, cardiomyopathy, renal damage, and impairment of spermatogenesis. Thus, with increasing abuse of METH worldwide, nephropathy, heart failure, and infertility are growing among users. Molecular and immunohistochemical examinations are necessary to clarify the adverse effects of METH on body systems at different doses and duration. Understanding METH pathogenesis can be useful for the development of novel therapeutic treatments for METH-addicted users.

## Authors’ Contributions

SA designed the study and investigated pathologic results, prepared the draft, and revised the manuscript; RKh evaluated the results; ShD prepared immunohistochemistry tissue sections; ML helped in the practical procedure.

## Ethical Statement

All animals received human care in compliance with the Guide for Care and Use of Laboratory Animals published by the National Institutes of Health (NIH publication No. 85-23, revised 1985). The study was approved by the Institutional Animal Care and Use Committee of our veterinary school.

## Conflicts of Interest

The authors report no conflicts of interest.
